# Exploration of the effect of sequence variations located inside the binding pocket of HIV-1 and HIV-2 proteases

**DOI:** 10.1038/s41598-018-24124-5

**Published:** 2018-04-10

**Authors:** Dhoha Triki, Telli Billot, Benoit Visseaux, Diane Descamps, Delphine Flatters, Anne-Claude Camproux, Leslie Regad

**Affiliations:** 1 Molécules thérapeutiques in silico (MTi), INSERM UMR-, S973 Paris, France; 2 IAME, UMR 1137, INSERM, Laboratoire de Virologie, Hôpital Bichât, AP-HP, Paris, France; 3 Université Paris Diderot, Sorbonne Paris Cité, Paris, France

## Abstract

HIV-2 protease (PR2) is naturally resistant to most FDA (Food and Drug Administration)-approved HIV-1 protease inhibitors (PIs), a major antiretroviral class. In this study, we compared the PR1 and PR2 binding pockets extracted from structures complexed with 12 ligands. The comparison of PR1 and PR2 pocket properties showed that bound PR2 pockets were more hydrophobic with more oxygen atoms and fewer nitrogen atoms than PR1 pockets. The structural comparison of PR1 and PR2 pockets highlighted structural changes induced by their sequence variations and that were consistent with these property changes. Specifically, substitutions at residues 31, 46, and 82 induced structural changes in their main-chain atoms that could affect PI binding in PR2. In addition, the modelling of PR1 mutant structures containing V32I and L76M substitutions revealed a cooperative mechanism leading to structural deformation of flap-residue 45 that could modify PR2 flexibility. Our results suggest that substitutions in the PR1 and PR2 pockets can modify PI binding and flap flexibility, which could underlie PR2 resistance against PIs. These results provide new insights concerning the structural changes induced by PR1 and PR2 pocket variation changes, improving the understanding of the atomic mechanism of PR2 resistance to PIs.

## Introduction

Type 1 and type 2 human immunodeficiency viruses (HIV-1 and HIV-2) are currently treated with the same therapeutic arsenal, which consists of drugs targeting integrase, reverse transcriptase, fusion protein and protease (PR). However, HIV-2 is naturally resistant to all non-nucleoside inhibitors of reverse transcriptase or fusion inhibitors. HIV-2 has also demonstrated reduced susceptibility to protease inhibitors (PIs)^1–8^. Recent *in vivo* studies have shown that HIV-2 does not produce a stronger virological response to a more recently developed class of integrase inhibitors than previously observed with PIs^9^. Moreover, in the case of resistance selection, HIV-2 quickly selects for mutations conferring cross-resistance to all PIs^10^. Therefore, novel and efficacious therapeutic agents for HIV-2 infection are urgently needed, as HIV-2 affects approximately 1 to 2 million patients, primarily in West Africa^11^.

PR is an effective therapeutic target for treating HIV infection because of its essential role in hydrolysing the viral Gag and Gag-Pol precursor polyprotein during infectious viral particle maturation. PR is an aspartic protease consisting of a symmetric homodimer with 99 amino acid residues in each monomer, including the catalytic triplet Asp-Thr-Gly, which is conserved in all aspartic proteases^12^. Currently, nine FDA (Food and Drug Administration)-approved PIs are available for HIV-1 therapy, including saquinavir (SQV), ritonavir, indinavir, nelfinavir, amprenavir (APV), lopinavir (LPV), atazanavir, tipranavir, and darunavir (DRV). Only three of these are commonly recommended for the treatment of HIV-2 infection: SQV, LPV, and DRV^1,3,4^. Greater understanding of the structural mechanisms underlying HIV-2 resistance to PIs is important for the development of new efficacious anti-HIV-2 drugs. To this end, several studies have compared HIV-1 and HIV-2 PR structures, hereafter referred to as PR1 and PR2, respectively. PR1 and PR2 share only approximately 50% of sequence identity but they exhibit a similar global fold, resulting in a small root mean square deviation (RMSD) value of approximately 1.0 Å^13–16^. The strongest structural differences were located at residues 15–20, 30–40, and 65–73^14,15,17^. Several studies have focused on the link between the differences of PR1 and PR2 affinity and certain amino acid changes (V32I, M46I, I47V, L76M, and V82I) between PR1 and PR2 binding sites. For example, Gustchina *et al*. (1991) compared PR1 structures with PR2 models and found that the differences in PR1 and PR2 affinity for two PIs were due in part to a V32I substitution^13^. Sardana *et al*. (1994) studied three other amino acid changes at positions 47, 76, and 82 and showed that these substitutions had minor effects on PI efficacy using enzymatic activity and inhibition studies^18^. Hoog *et al*. (1995) confirmed the partial effect of amino acid changes at positions 47 and 82 on the specificity of a peptide analogue inhibitor for PR2 and also showed the involvement of substitution at position 32^19^. More recently, Tie *et al*. (2012) compared PR1 mutants containing these three mutations (V32I, I47V, and V82I) with PR1 and PR2 wild-type structures in terms of global biochemical properties and their interactions with APV^16^. These authors showed that, despite the PR1 mutant structure being similar to the wild-type PR1 structure, the PR1 mutant interaction with APV was similar to that of PR2, as was its global biochemical properties. The importance of these amino acid changes was also confirmed by Raugi *et al*. (2016), who reported the opposite observation by incorporating four HIV-1-like mutations, I32V, V47I, L76M, and I82V, into a wild-type PR2. This PR2 mutant exhibited susceptibility to all PIs perfectly equivalent to PR1^3^. In this last study, the authors also showed that mutations in the binding site induced only small rearrangements, modifying the internal interactions between PR2 and DRV compared to the PR1-DRV complex. All of these studies have focused on the analysis of substitution effects on the PR structure complexed with only one FDA-approved drug, and they have not considered the diversity in PIs. Moreover, they have highlighted the effects of amino acid changes on the different interactions established between PR and the drug but they have provided little information regarding the structural changes in PR itself induced by these mutations. Thus, the structural characteristics leading to the differing patterns of PI sensitivity have not been adequately explored to date.

Currently, there are approximately 600 PR1 structures and 19 PR2 structures available in the Protein Data Bank (PDB)^20^. These structures correspond to different forms (bound or unbound) complexed with different ligands. Performing a systematic comparison of the ligand-binding sites extracted from diverse PR1 and PR2 structures could provide information concerning the effects of variations between PR1 and PR2 sequences, regardless of the complexed ligands that improve the understanding of PR2 resistance to PIs.

In this study, we performed the first systematic comparison of PR1 and PR2 binding sites considering unbound and bound PR structures and a variety of ligands, including FDA-approved drugs and chemical small molecules. PR binding sites were first estimated by extracting pockets from 24 crystallographic structures of PR1 and PR2, both in unbound forms and complexed with 12 different ligands. Considering all these ligands during the pocket estimation yielded pockets independent of the co-crystallized ligand that allowed comparisons of pockets complexed with diverse ligands. Second, we performed a systematic comparison of the PR1 and PR2 pockets described using a large set of descriptors and statistical approaches. Our results showed that PR1 and PR2 pockets exhibit different physicochemical properties; PR2 pockets are more hydrophobic, with fewer hydrophobic residues and increased numbers of tiny residues with more oxygen atoms and fewer nitrogen atoms than PR1 pockets. Finally, the links between the highlighted PR1 and PR2 pocket differences and pocket sequence variations were analysed to provide information concerning the effects of amino acid changes between PR1 and PR2 sequences in the binding site, regardless of the bound ligands. These results showed that sequence changes located in the PR pockets are responsible for the physicochemical differences in PR1 and PR2 pockets. In addition, we observed that several differences were explained by structural changes observed in main-chain atoms of substituted residues and in residue 45 of chain B, named 45_B, a non-substituted residue. The structural comparison of PR1 mutants and PR1 and PR2 wild-type structures suggested a cooperative movement induced by the substitutions V32I and L76M, which induces the structural change observed at residue 45_B in PR2. We speculated that the particular conformation of residue 45_B in PR2 and the substitutions M46I and I47V could modify the flexibility of PR2, impacting PI binding. Our analysis of PR1 and PR2 pockets improves the understanding of roles of sequence variations between PR1 and PR2 pockets in the structural changes observed in the PR2 pocket that could be involved in the PR2 resistance to PIs. In addition, our results provide new insight that could be exploited in the design of more efficient inhibitors for PR2.

## Results

### PR, ligand and pocket set description

#### PR structure set

**Figure 1:**
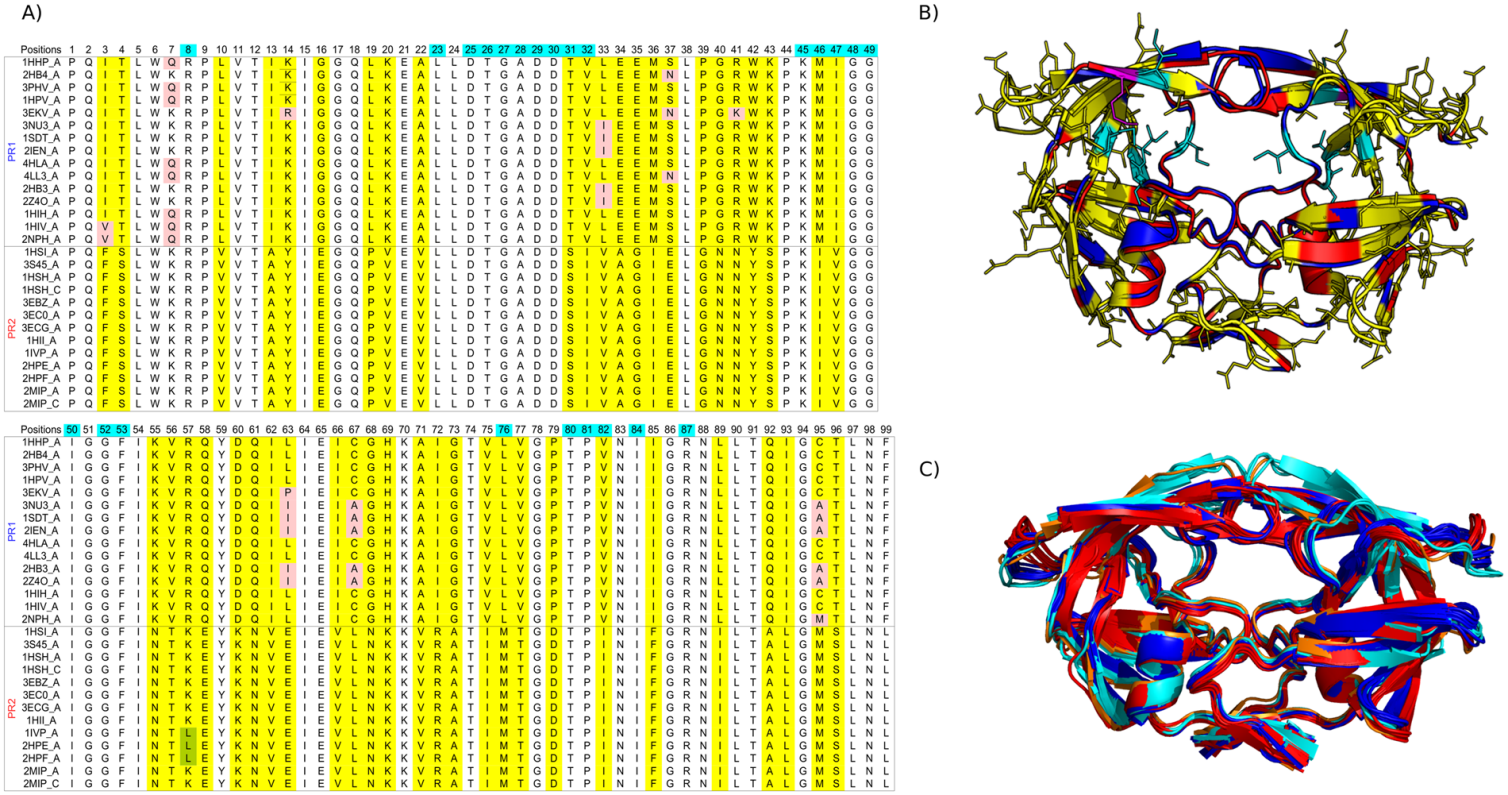
The PR set. (**A**) Multiple sequence alignment of the 28 PR1 and PR2 sequences. Highlighted residues correspond to mutated residues between PR sequences: mutations between PR1 sequences are highlighted in pink, mutations between PR2 sequences are highlighted in green, amino acid changes between PR1 and PR2 sequences are highlighted in yellow. Residues located in the binding pockets are over-lined in blue. (**B**) Superimposition of PR1 (PDB code: 1HPV, coloured blue) and PR2 (PDB code: 3EBZ, coloured red) structures. Substitutions between PR1 and PR2 sequences are displayed as sticks and coloured yellow, and those located in the binding pocket are coloured cyan. Residue 45_B is displayed as sticks and coloured magenta. (**C**) Superimposition of the 28 PR. Unbound and bound PR1 structures are coloured in cyan and blue, respectively. Unbound and bound PR2 structures are coloured in orange and red, respectively.

**Figure 2:**
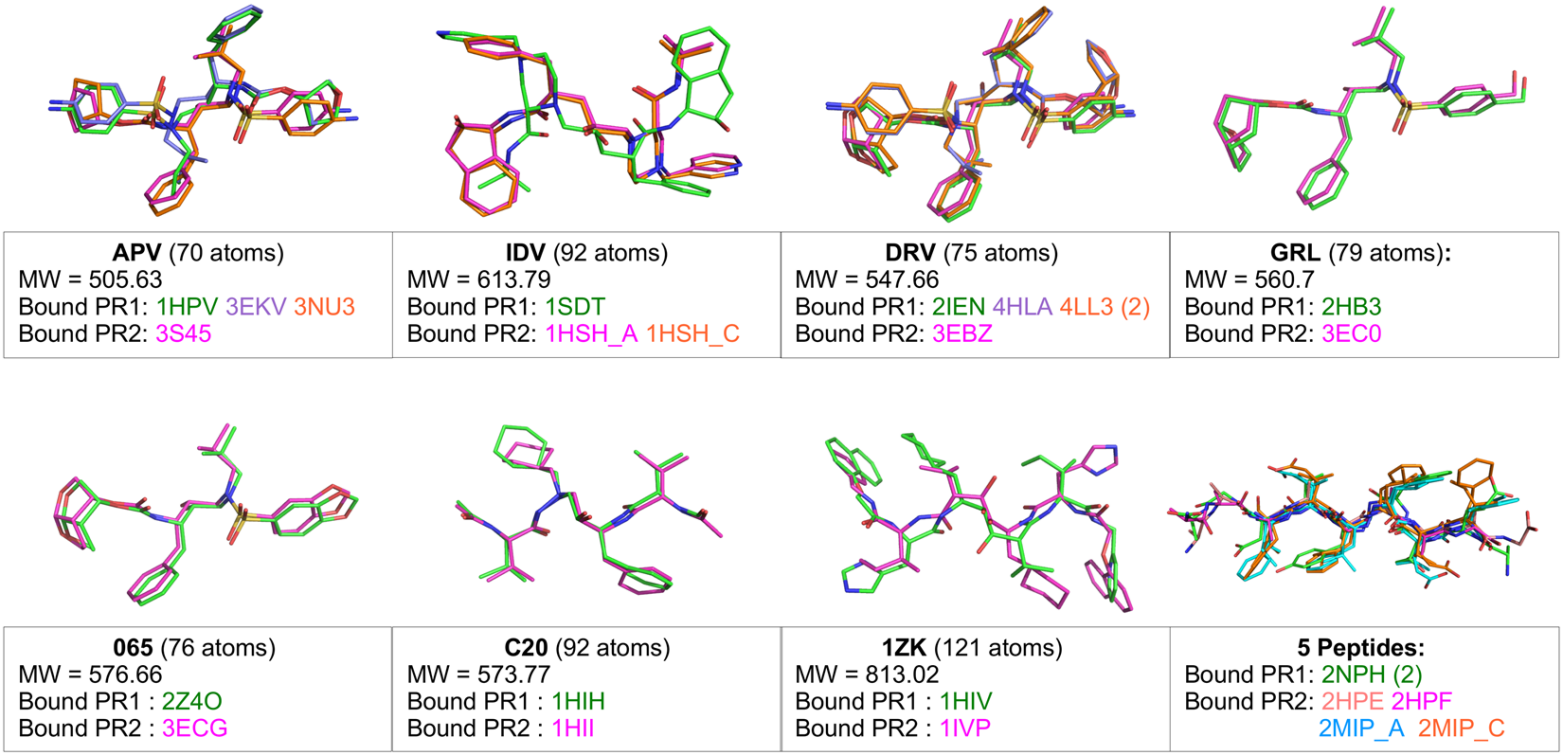
The ligand set. Structure of ligands extracted from the 24 superimposed PR complexes. The size (in atoms) and the molecular weight (MW; g/mol) are indicated for each ligand. In addition, the PDB code of the complex from which each ligand was extracted is indicated.

In this study, we used a set of 28 PR three-dimensional structures containing 15 PR1 and 13 PR2 dimers, referred to as the PR set (S1 Table and Fig. 1). This set is composed of 4 unbound PR and 14 PR complexed with 8 diverse ligands. For each ligand, we have at least one structure of PR1 and PR2 complexes (S1 Table and Fig. 2). Figure 1A shows the multiple alignment of amino acid sequences extracted from the PR set. There were nine mutated positions between the 15 PR1 sequences. Five of these mutations (Q7K, L33I, L63I, C67A, and C95A) correspond to classical experimental mutations introduced to minimize autoproteolysis and to prevent cysteine-thiol oxidation of PR1^21^. One mutation (K57L) was observed among the 13 PR2 sequences. Fifty amino acid changes were identified between PR1 and PR2 sequences, including the five PR1 positions mutated due to the autoproteolysis. These amino acid changes were located throughout the sequence and structure (Fig. 1A,B).

The PR set includes four unbound structures—i.e., not complexed with a ligand, and 24 structures bound to different ligands. Figure 1C shows the superimposition of the 28 total PR structures. The PR1 and PR2 structures exhibit a similar fold that results in a weak average main-chain RMSD (1.20 ± 0.57 Å), in agreement with^13–16^. The largest structural differences between the two PRs are located at residues in the outer loops, particularly in the elbow and flaps, known to be highly mobile. As previously shown, the four PR unbound structures have flap regions in semi-open conformations, while the bound PR structures exhibit flap regions in closed conformations (Fig. 1C).

#### PR ligand set

The 24 bound PR structures were complexed with 12 different ligands: three FDA-approved drugs, four chemical compounds and five peptides (Fig. 2). This ligand set is diverse in terms of size, ranging from 54 atoms for the peptide extracted from the 2MIP (PDB code) structure to 70 atoms for APV extracted from the 3S45 (PDB code) structure (Fig. 2). Certain of these ligands are flexible. This flexibility resulted from different sources. We observed small shifts in the superimposed PR1 and PR2 ligands, such as for the GRL and 065 ligands (Fig. 2). Several ligands had the capacity to bind PR with different orientations in both PR1 and PR2, such as IDV and C20, or in one PR, such as APV and DRV, which bind to PR1 with two orientations (Fig. 2).

We used all these superimposed ligands to estimate PR pockets that allowed us to consider the ligand diversity and flexibility.

#### PR pocket set

**Figure 3:**
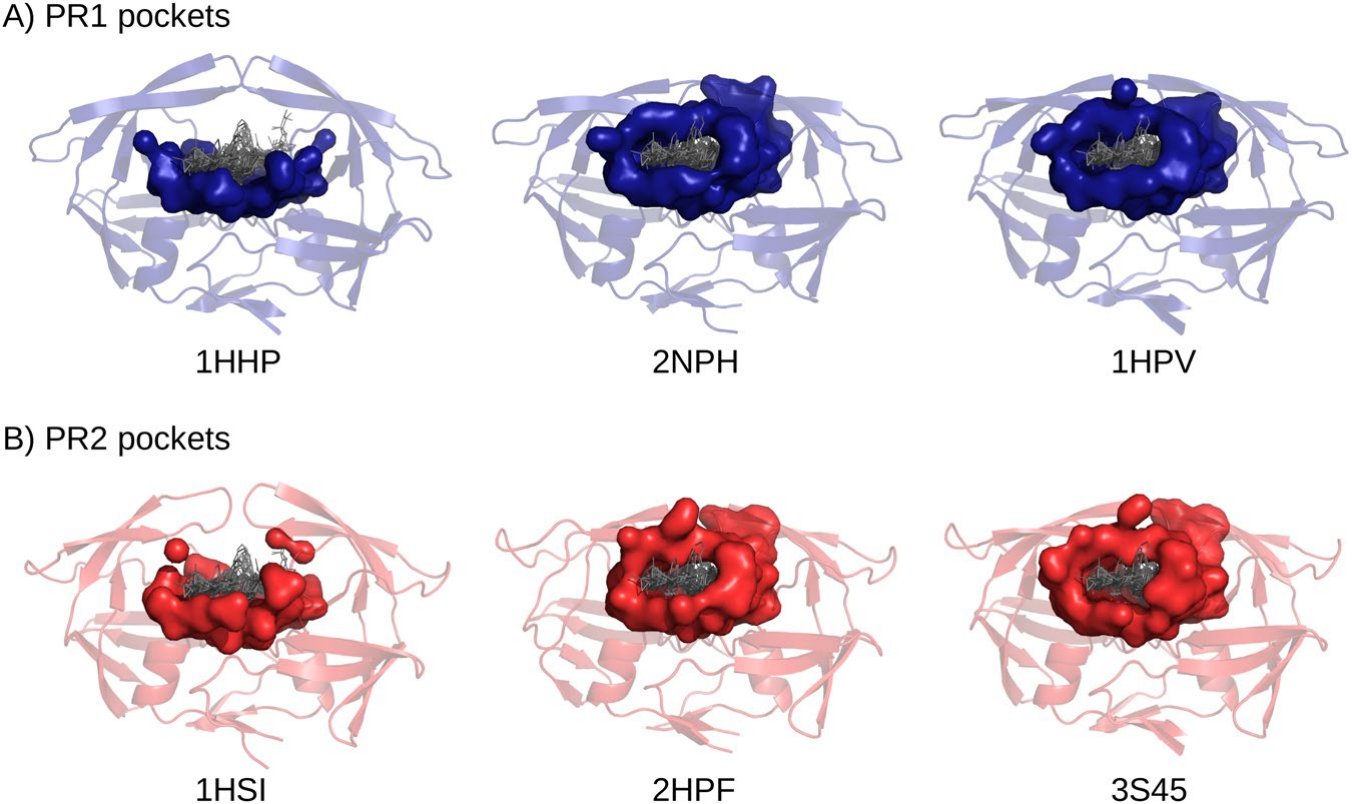
Visualization of pockets extracted from three PR1 structures (**A**) and three PR2 structures (**B**). Left, pockets corresponding to pockets extracted from PR unbound forms (from 1HHP (PDB code) for PR1 and 1HSI (PDB code) for PR2); middle, pockets corresponding to pockets extracted from PR structures complexed with a peptide (from 2NPH (PDB code) for PR1 and from 2HPF (PDB code) for PR2); and right, pockets corresponding to pockets extracted from PR structures complexed with APV (from 1HPV (PDB code) for PR1 and from 3S45 (PDB code) for PR2). PR structures are shown as cartoons, and pockets are shown as surface. Pockets were extracted based on the proximity to all ligands of the set (see Methods).

Ligand-binding pockets were extracted from each PR structure (PR unbound and bound) by determining their atoms situated in proximity to the ensemble of all superimposed ligands of the set (see Material & Methods). This resulted in a set of 28 pockets extracted from the PR set, called the PR pocket set. Each pocket corresponds to a larger region capable of binding all ligands of the dataset. Figure 3 shows pockets extracted from six PRs (3 PR1 and 3 PR2 structures): two were extracted from unbound structures, hereafter referred to as unbound pockets, and four were extracted from bound PR structures, hereafter referred to as bound pockets. Unbound pockets had different geometries compared to bound pockets. Indeed, the pocket lid, composed of flap atoms, was missing in the unbound pocket. This is explained by the fact that the flap regions in the PR unbound structures were in an open conformation, whereas they had a closed conformation in bound PR structures. We also observed that bound pockets exhibited similar geometries regardless of the complex from which they were extracted. Indeed, to estimate pockets, we used all ligands extracted from the PR set and not the co-crystallized ligand classically utilized^22–24^. This estimation induced that the estimated pocket shape was independent of the co-crystallized ligand but was built by taking into account all bound ligands, their diversity and their flexibility.

**Figure 4:**
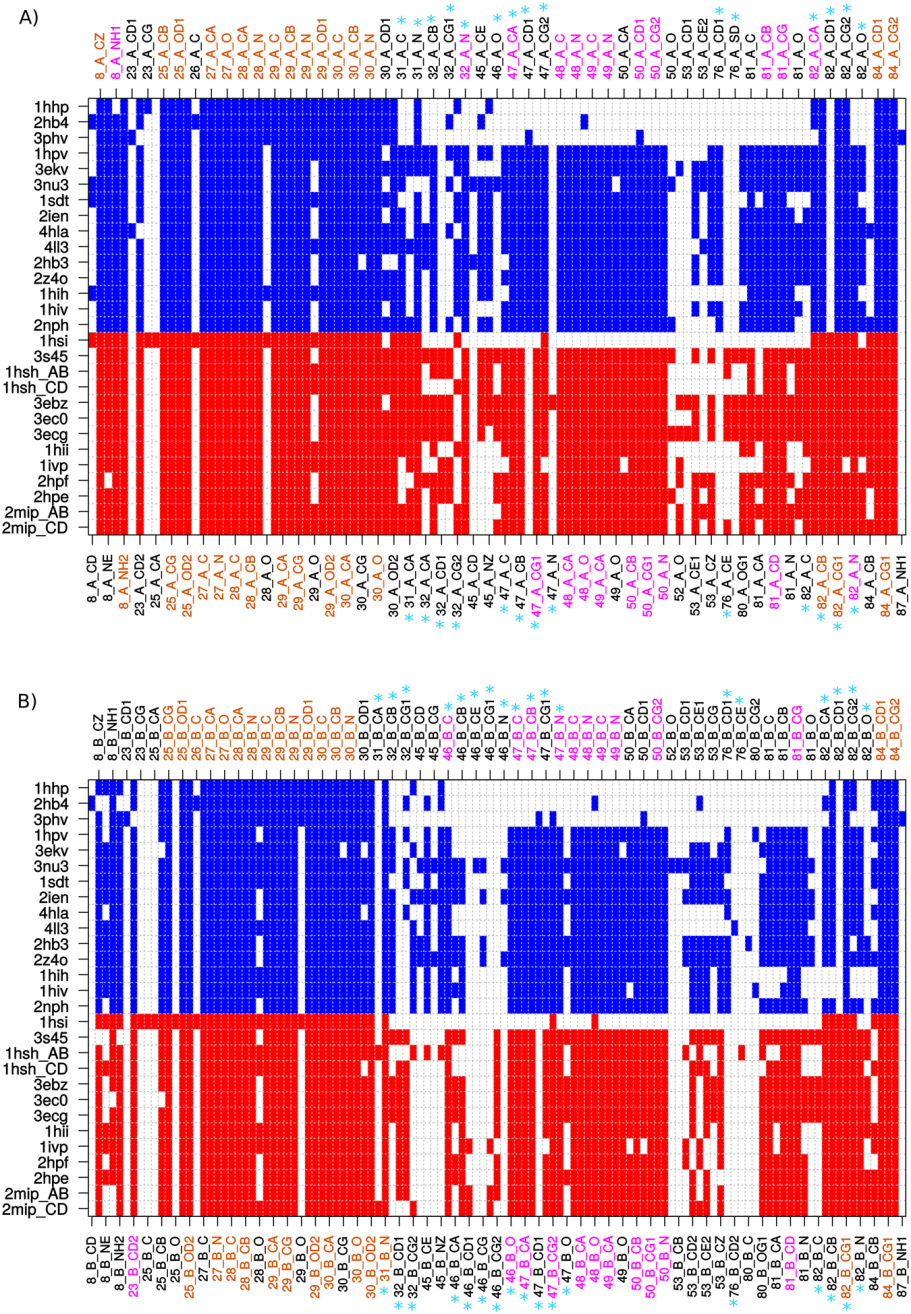
PR1 and PR2 atoms of chains A (**A**) and B (**B**) involved in pockets. Pocket atoms extracted from PR1 and PR2 structures are coloured blue and red, respectively. Pocket atoms of substituted residues between PR1 and PR2 are indicated in cyan stars. Atoms observed in all pockets are highlighted in orange. Atoms observed in all bound pockets are highlighted in magenta.

PR pockets contained 38 ± 5.02 residues on average, and the consensus pocket contained 48 different residues, referred to as pocket residues. Of these 48 pocket residues, six were substituted between PR1 and PR2 pockets: T31S, V32I, M46I, I47V, L76M, and V82I. In terms of atoms, PR pockets contained 151 atoms (±25) on average and approximately as many side-chain atoms as main-chain atoms (55% and 53% for PR1 and PR2, respectively). A total of 220 different atoms, named pocket atoms, were observed in all PR pockets. Their distribution in the two PR pocket sets is shown in Fig. 4. Among these atoms, 27% (60 atoms) were found in all PR pockets. These conserved atoms corresponded as much to the main-chain atoms (48%) as to the side-chain atoms (52%) and were mainly located around the catalytic site. Their conservation in all PR pockets suggests that these atoms are important for protease function and ligand binding. In addition, we identified 43 atoms conserved in all bound pockets but not present in unbound pockets. Most of these were located in flaps; two (32_A_N [azote atom of residue 32 in chain A] and 23_B_CD2) were adjacent to the catalytic site, and seven (atoms of residues 81 and 82) were adjacent to and within the wall region. These results suggest that the atoms only present in bound pockets may be involved in PR deformation upon ligand binding and flap movement. These results highlight that structural changes induced by ligand binding occur in both flap and wall regions.

### PR1 and PR2 pocket characterization and comparison

#### PR1 and PR2 pocket druggability

To explore if the decreased PI susceptibility of PR2 could be explained by a different pocket ability to bind drug-like molecules, hereafter referred to as the pocket druggability, we computed the druggability score of each PR1 and PR2 pocket using the PockDrug webserver^22,25^. All PR pockets were predicted to be druggable, with a similar average high score (0.90 ± 0.09 and 0.95 ± 0.03 for PR1 and PR2, respectively; Student test p-value = 0.07). Thus, the weaker sensitivity of PR2 to PIs relative to PR1 is not due to differences in druggability between their pockets. To assess the role of pockets in the decreased PI susceptibility of PR2, we performed an in-depth analysis of the physicochemical and geometric properties of PR1 and PR2 pockets.

#### Comparison of PR1 and PR2 pocket properties

**Figure 5:**
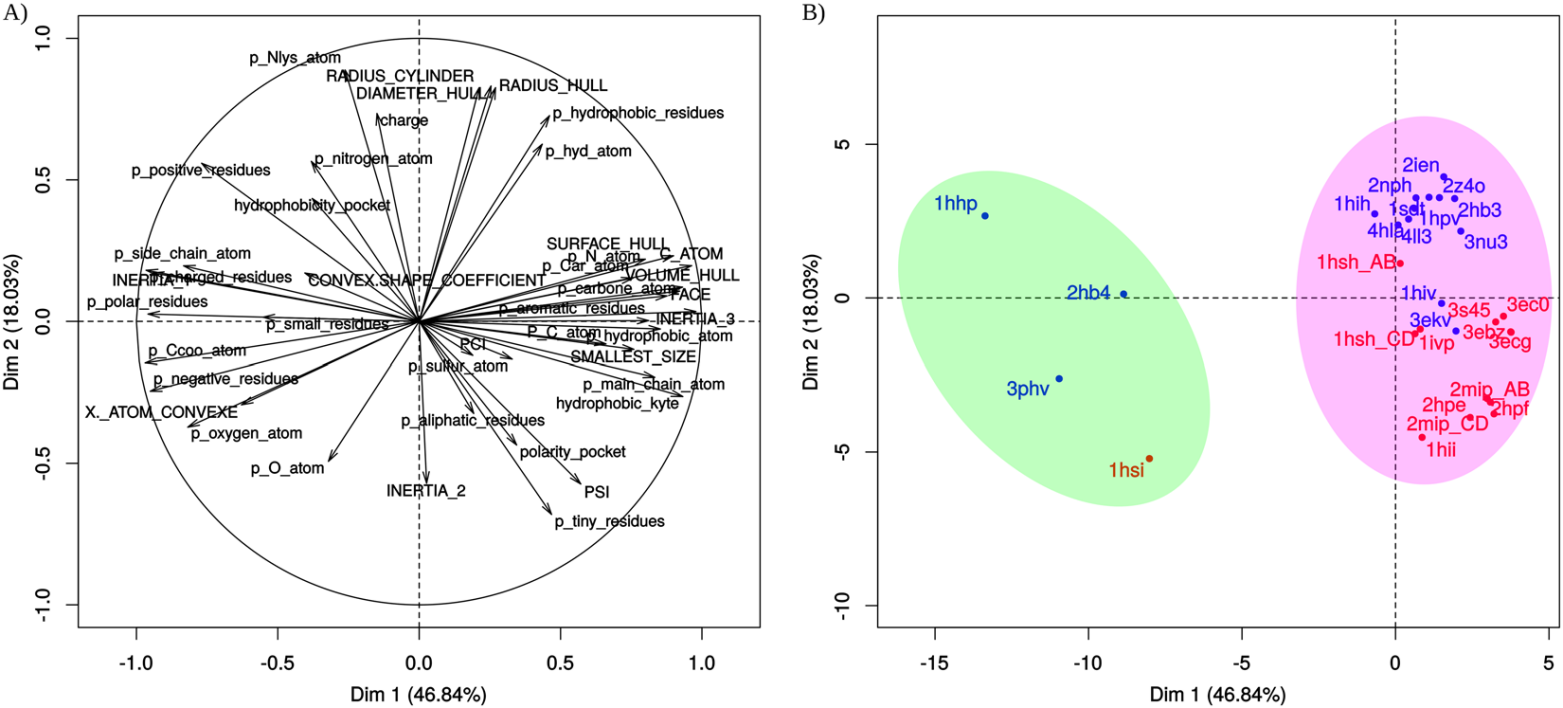
Projection of the 42 descriptors (**A**) and of the 28 PR pockets (**B**) in the first PCA plane. The first PCA plane explained 65% of the data variability. PR1 and PR2 pockets are coloured in blue and red, respectively. The green ellipse highlights pockets extracted from PR unbound structures, and the pink ellipse highlights pockets extracted from bound PR structures.

The 28 PR pockets were described using a large set of 42 physicochemical and geometrical descriptors (see S1 Appendix). We first analysed the space sampled by the 28 pockets by principal component analysis (PCA), based on the descriptors (Fig. 5). The PR unbound pockets were clearly separated from the bound PR pockets in the first PCA plane, indicating that they have different properties. The interpretation of the first PCA dimension in terms of descriptors showed that unbound pockets tended to be smaller and more convex (higher X._ATOM_CONVEXE descriptor values) with more polar and charged residues than bound pockets (Fig. 5A). These differences between unbound and bound pockets stemmed from the different conformations of the flap regions in PR unbound and bound structures (Fig. 3).

Regarding bound pockets, most PR2 pockets were easily differentiated from PR1 pockets on the PCA second axis (Fig. 5A). The second PCA dimension indicates that PR2 pockets are smaller than those of PR1, according to the *RADIUS_CYLINDER*, *RADIUS_HULL* and *DIAMETER_HULL* descriptors. PR2 pockets also contained higher numbers of tiny and less hydrophobic residues (*p_tiny_residues* and *p_hydrophobic_residues* descriptors) but were globally more hydrophobic (higher values of the *hydrophobicity_kyte* descriptor) than PR1 pockets. Thus, we conclude that even though the main difference was observed between unbound and bound pockets, several of the 42 pocket descriptors were able to discriminate between PR1 and PR2 bound pockets. In subsequent experiments, we focused only on the characterization and analysis of the 24 PR1 and PR2 bound pockets.

**Figure 6:**
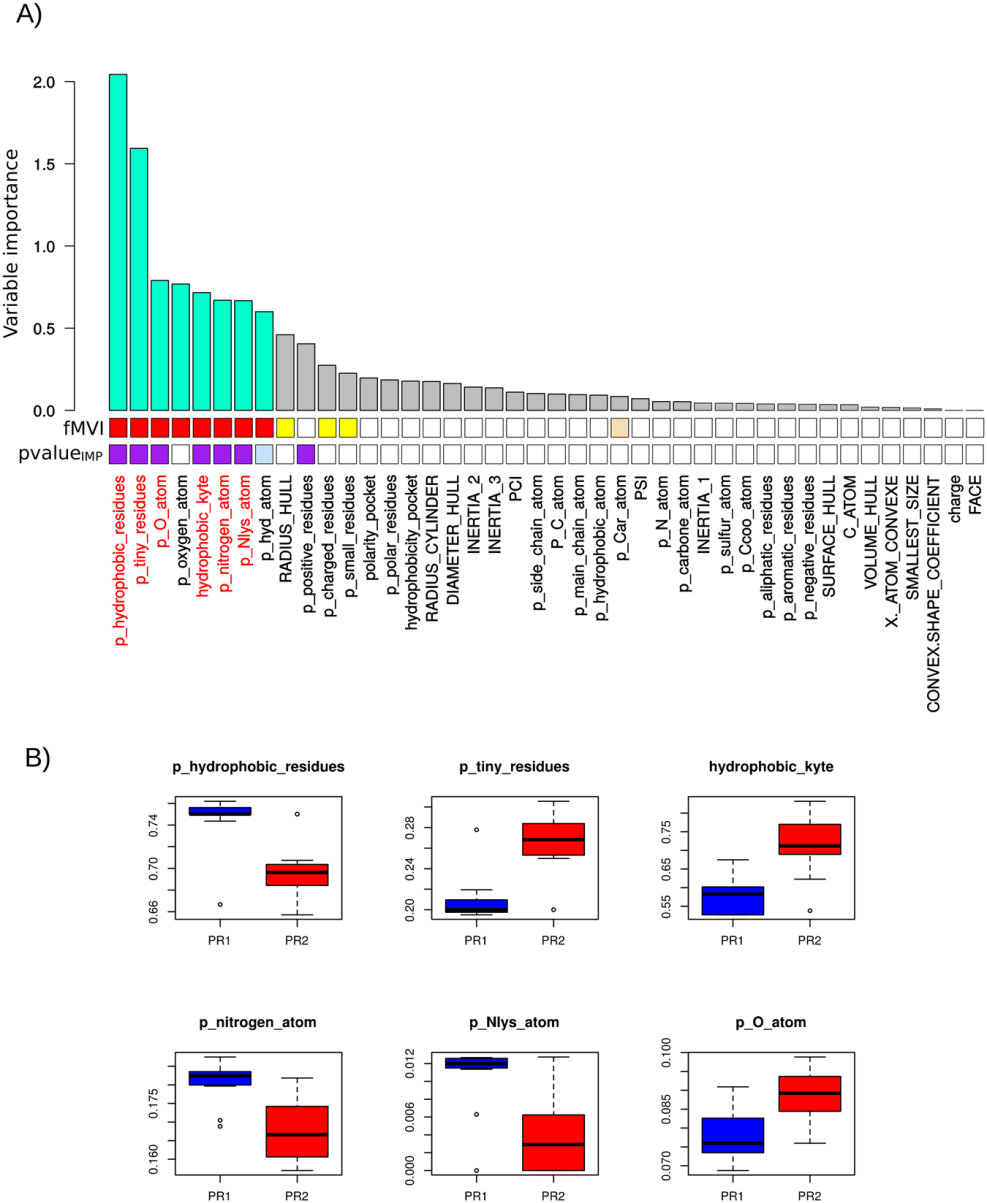
Physicochemical and geometric differences between PR1 and PR2 pockets. (**A**) RF importance scores of the 42 pocket descriptors in the *RF*_*PR1-PR2*_ model. The eight descriptors selected as important for the RF model (with an importance score higher than 0.5) are coloured cyan. *fMVI* corresponds to the proportion of RF models where a descriptor was selected as one of the ten most important variables. This value was computed from a simulation of 500 RF models computed using the same data and the same parameters as those used to compute the *RF*_*PR1-PR2*_ model (see S2 appendix). *fMVI* values between 0% and 20% are shown in white; *fMVI* values between 20% and 40% are shown in wheat; *fMVI* values between 40% and 60% are shown in yellow; *fMVI* values between 60% and 80% are shown in orange; *fMVI* values between 80% and 90% are shown in magenta; and *fMVI* values higher than 90% are shown in red. *pvalue*_*IMP*_ corresponds to the importance score p-value, computed as a fraction of null importance (importance computed from *RF*_*permuted*_) scores that were more extreme than the importance score in the *RF*_*PR1-PR2*_ model. All *pvalue*_*IMP*_ smaller than 0.06 are coloured light blue and *pvalue*_*IMP*_ values smaller than 0.05 are coloured purple. (**B**) Distribution of the six selected descriptors in PR1 (blue) and PR2 (red) pocket sets.

To identify the physicochemical and geometric differences between PR1 and PR2 bound pockets, we selected the properties most able to discriminate between PR1 and PR2 pockets. To do so, we trained a random forest (RF) model on the 24 bound pockets characterized by the set of 42 descriptors. The obtained RF model, named the *RF*_*PR1-PR2*_ model, exhibited robust performances with a very low error rate, confirming its ability to discriminate between PR1 and PR2 bound pockets. The S2 Appendix shows a precise description of the *RF*_*PR1-PR2*_ model. Figure 6A shows the involvement of the 42 descriptors in the *RF*_*PR1-PR2*_ model, quantified according to their importance score. The higher the score is, the more important the descriptor for differentiating between PR1 and PR2 pockets.

We observed that descriptors having the largest importance scores corresponded to physicochemical descriptors, indicating that PR1 and PR2 pockets exhibited more physicochemical differences than geometric differences (Fig. 6A). Among the 42 pocket descriptors, six were significant for separating PR1 and PR2 pockets (they had an importance score greater than 0.5 and a *pvalue*_*IMP*_ smaller than 0.05, Figs 6A and S2 Appendix). They characterized the pocket hydrophobicity (*p_hydrophobic_residues* and *hydrophobibity_kyte*), the composition of certain atoms (oxygen [*p_oxygen_atom*] and nitrogen [*p_nitrogen_atom* and *p_Nlys_atom*]) and tiny residues (*p_tiny residues*). Two of them, characterizing the pocket composition of hydrophobic residues (*p_hydrophobic_residues*) and tiny residues (*p_tiny residues*), were the most discriminatory between PR1 and PR2 pockets, in agreement with the PCA results. Figure 6B shows the distribution of these six descriptors in PR1 and PR2 pockets. These findings indicate that PR2 pockets are globally more hydrophobic than PR1 pockets, according to their kyte scores (*hydrophobibity_kyte*), with fewer hydrophobic residues and more tiny residues. Moreover, PR2 pockets contain more oxygen atoms and fewer nitrogen atoms than PR1 pockets.

**Figure 7:**
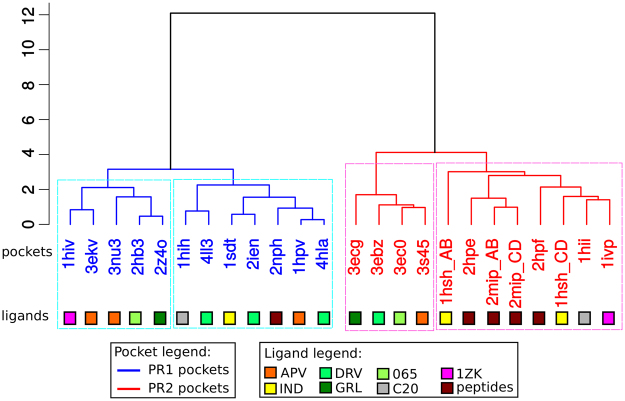
Hierarchical classification of PR pockets based on the six descriptors selected as important for discriminating PR1 and PR2 pockets. PR1 and PR2 pockets are coloured blue and red, respectively. Coloured squares indicate the ligand co-crystallized with the pockets.

A hierarchical classification of the 24 bound PR1 and PR2 pockets was performed using the six most discriminatory descriptors (Fig. 7). As expected, the classification identified two PR pocket clusters; the first cluster contained only PR1 pockets, and the second cluster contained only PR2 pockets. Within these two PR pocket clusters, several PR pocket sub-clusters were identified. The two PR2 pocket sub-clusters were differentiated earlier in the classification process compared to the two PR1 pocket sub-clusters, indicating that the PR2 pockets are more heterogeneous than the PR1 pockets in terms of these six descriptors. By comparing the bound ligands in each PR pocket sub-cluster, we noted that the two PR1 pocket sub-clusters were not linked to the co-crystallized ligands: two PR1 pockets bound to similar ligands were not always found in the same sub-cluster, such as PR1 pockets bound to DRV or its derivatives (e.g., GRL 065). In contrast, PR2 pockets bound to DRV or its derivatives constituted one sub-cluster. The PR2 pocket bound to APV completed this PR2 pocket sub-cluster, indicating that the PR2 pockets binding DRV and APV exhibited similar physicochemical properties, whereas the corresponding PR1 pockets were more dissimilar.

### Association of PR1 and PR2 pocket differences with their sequence variation

#### Analysis of the impact of sequence variations between PR pockets on their properties

PR pockets exhibited six amino acid changes (T31S, V32I, M46I, I47V, L76M and V82I). To link these sequence variations with the physicochemical differences between PR1 and PR2 pockets, we analysed and compared the physicochemical properties of each changed residue. For example, the substitution of a threonine (a hydrophobic residue) by a serine (a tiny residue) at position 31 in PR2 resulted in PR2 pockets having fewer hydrophobic residues and more tiny residues than PR1 pockets. In addition, at positions 32, 46 and 82 of PR2, valine or methionine residues in PR1 were substituted by an isoleucine (more hydrophobic) in PR2. These amino acid changes induced that PR2 pockets are globally more hydrophobic than PR1 pockets. Thus, these sequence variations were directly responsible for the observed physicochemical differences between PR1 and PR2 pockets.

To understand the other physicochemical changes between the two PR pockets, we compared the atomic composition of the PR1 and PR2 pockets (Fig. 4). As expected, we observed differences in side-chain atoms of substituted residues between PR1 and PR2 pockets. Indeed, side-chain atom changes of substituted pocket residues resulted in several atoms were observed in only one PR type. For example, the substitution of the threonine at position 32 by a serine resulted in atom CD1 (PDB atom name) of residue 32 of both chains, denoted as 32_A/B_CD1, were observed in only PR2 pockets. In addition, atoms 46_B_CG2 and 82_A/B_CD1 were observed only in PR2 pockets, whereas atoms 47_A/B_CD1 and 76_A/B_CD1 were observed only in PR1 pockets. Surprisingly, we observed differences in the composition of PR1 and PR2 pockets in terms of the main-chain atoms of substituted residue pockets. Three main-chain atoms of substituted residues (31_A_CA, 46_A_O, and 82_B_O) were more frequent in PR2 than in PR1 pockets (Fig. 4). These main-chain atom changes between PR1 and PR2 pockets were captured by physicochemical descriptors; PR2 pockets contain more oxygen atoms than PR1 pockets. We speculate that the composition changes between PR1 and PR2 pockets in main-chain atoms of substituted residues (e.g., 31, 46, and 82) are directly induced by T31S, M46I, and V82I amino acid changes. Thus, we suggested that these structural changes could modify PI binding in PR2.

**Figure 8:**
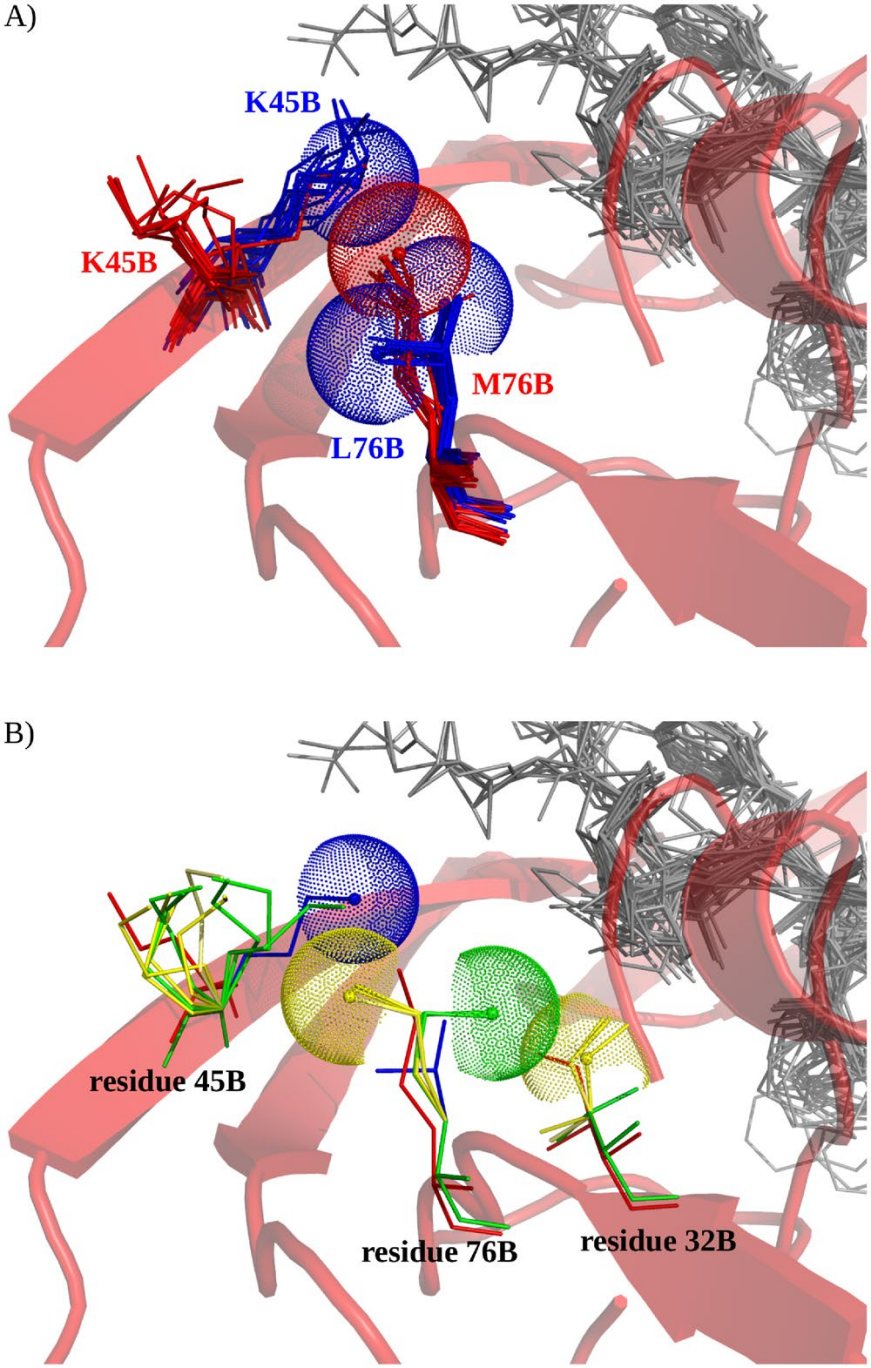
Structural differences of residues 45_B in PR1 and PR2 pockets. (**A**) Conformation of residues 45_B and 76_B in all PR1 and PR2 pockets. These residues are displayed as sticks and coloured blue for PR1 and red for PR2. Blue dots represent the van der Waals spheres of the NZ atom of PR1 lysine 45_B and the CD1 and CD2 atoms of PR1 leucine 76_B. Red dots represent the van der Waals spheres of the CE atom of PR2 methionine 76_B. PR2 (PDB code: 3S45) is displayed as a cartoon and coloured red. All ligands of the PR set and used to estimate the pockets are shown in sticks mode and coloured in grey. (**B**) Conformation of residues 45_B, 76_B and 32_B of PR1 (coloured blue, PDB code: 1HPV), PR2 (coloured red, PDB code: 3S45), PR1_L76M_ mutants (coloured green) and PR1_V32I+L76M_ mutants (coloured yellow). These residues are shown in sticks mode. Green dots represent the van der Waals sphere of the M76_B_CE atom of PR1_L76M_ mutant number 2. Cyan dots represent the van der Waals sphere of the I32_B_CE atom of PR1_V32I+L76M_ mutant number 4. PR2 (PDB code: 3S45) is shown in a cartoon mode and coloured red. All ligands of the PR set used to estimate pockets are displayed in sticks mode and coloured grey.

More surprisingly, differences in the PR1 and PR2 atom composition were also observed for two side-chain atoms (CE and NZ, PDB atom name) of non-substituted residue 45 in both chains (Fig. 4). These atoms were partially (chain A) or totally (chain B) absent in PR2 pockets. This absence is explained by the fact that residue 45 exhibits different conformations in PR1 and PR2 pockets; in PR1 pockets, the side-chain of residue 45 points towards the pocket inside, whereas in PR2, it points to the protein surface (Fig. 8A). The lack of a lysine at residue 45 in most PR2 pockets explains the lower levels of lysine nitrogen atoms in PR2 pockets relative to PR1 pockets.

#### Link between the substitutions located in the PR pocket and the structural changes of residue 45_B

**Figure 9:**
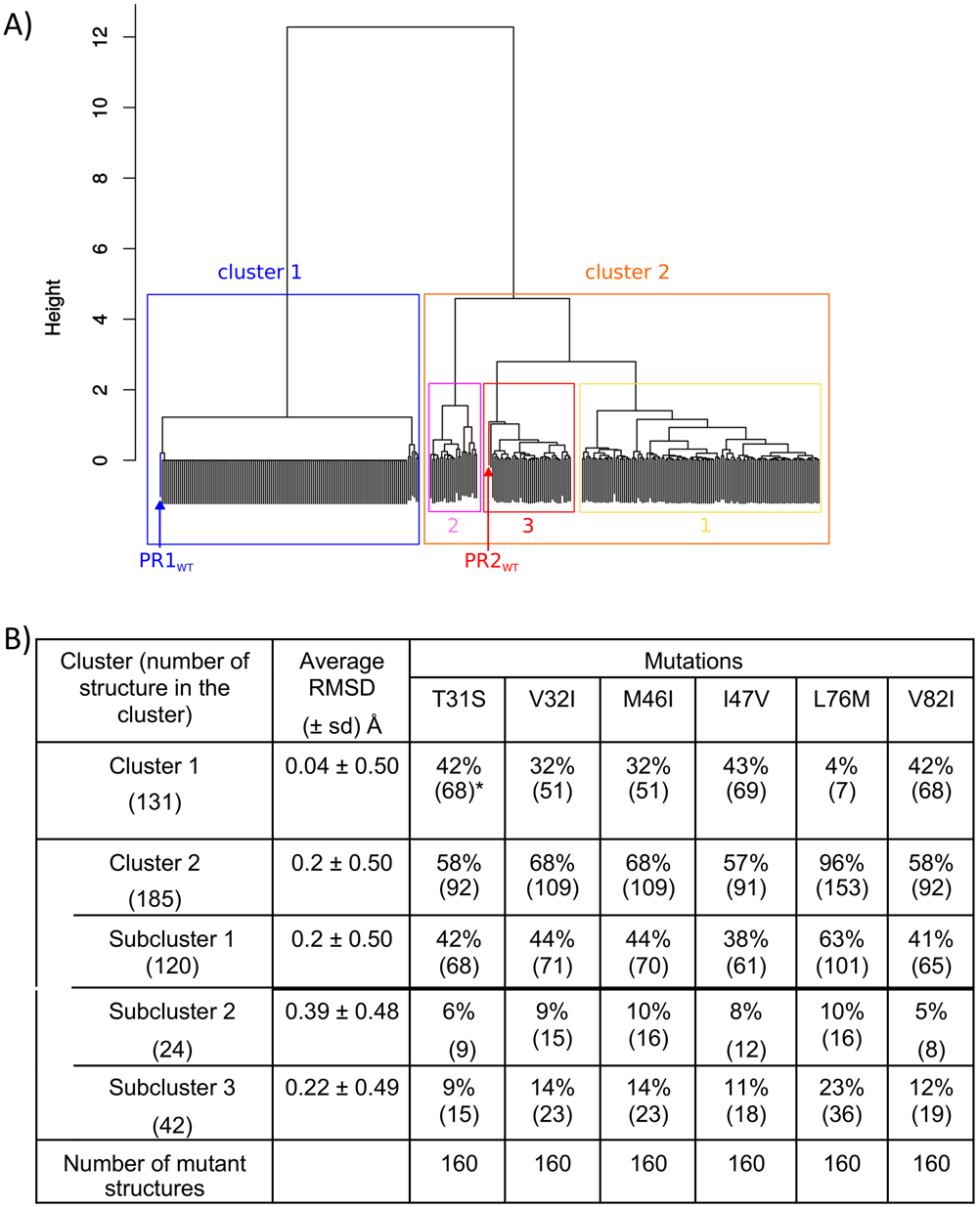
Analyse of the link between structural changes of residue 45_B between PR1 and PR2 structures and the substitutions observed between PR1 and PR2 pockets. (**A**) Hierarchical classification of 1 PR1 wild-type structure (PDB code: 1HPV), 1 PR2 wild-type structure (PDB code: 3S45), and the 315 PR1 mutant structures modelled using FoldX^26^ software. The classification was based on the RMSD distance computed on the residue 45_B between all structure pairs and the Ward aggregation method. The classification was first split in two clusters. Cluster 2 was then split into three sub-clusters. (**B**) Repartition of the six substitutions observed between PR1 and PR2 pockets in each cluster extracted from the classification based on the 45_B conformation. *Among the 160 mutant structures with the mutation T31S, 68 are in cluster 1 (42% = 68/160).

Our results showed that non-substituted residue 45_B exhibits different conformations in PR1 and PR2 (Fig. 8A), which induced a change in PR2 pocket composition relative to the PR1 pocket. In this section, we studied the link between structural changes observed at residue 45_B between PR1 and PR2 structures and the six substitutions occurring between PR1 and PR2 pockets (T31S, V32I, M46I, I47V, L76M, and V82I). For this purpose, we modelled 63 PR1 mutants containing one to six pocket substitutions using the FoldX software^26^. For each mutant, we built five mutant structures that lead to a set of 315 PR1 mutant structures (see Material & Methods). We compared the conformation of residue 45_B in one PR1 (PDB code: 1HPV) and one PR2 (PDB code: 3S45) wild-type structures and in the 315 PR1 mutant structures. To do so, we computed a hierarchical classification of the 315 PR1 mutant structures, the wild-type PR1 and PR2 structures based on the RMSD computed between residue 45_B of all structures (see Method). Figure 9A presents the obtained dendrogram. As expected, the wild-type PR1 and PR2 structures are clearly separated in the classification. Indeed, the obtained classification was first divided into two clusters: 130 PR1 mutant structures and the wild-type PR1 structure constitute cluster 1, whereas 185 PR1 mutant structures and the wild-type PR2 structure constitute cluster 2. We noted that cluster 1 contains structures with very similar conformations of residue 45_B (average RMSD = 0.04 ± 0.5 Å, Fig. 9B). Residues 45_B in these mutant structures have a conformation closer to its in the wild-type PR1 structure than in the wild-type PR2 structure. This indicates that mutations occurred in these mutant structures induce no or very small structural deformation of residue 45_B. In contrast, structures of cluster 2 exhibit more diverse conformations of residue 45_B, with an average RMSD of 0.2 Å (±0.5 Å, Fig. 9B). The conformation of residue 45_B in the PR1 mutant structures of cluster 2 is closer to its in the wild-type PR2 structure than in the wild-type PR1 structure. Thus, mutations occurred in the mutant structures of cluster 2 induce large structural deformation of residue 45_B. Figure 9B summarizes the frequency of each mutation in the four clusters. We observed that three mutations (V32I, M46I, and L76M) did not equitably sample clusters 1 and 2, particularly mutation L76M. Indeed, among the 160 mutant structures with the mutation L76M, 96% were grouped in cluster 2 and only seven mutant belong to cluster 1 (Fig. 9B). The three other mutations sample fairly the two groups (Fig. 9B).

Cluster 2 was then split into three sub-clusters (Fig. 9A). The sub-cluster 3 groups the wild-type PR2 structure with 41 PR1 mutant structures corresponding to 28 different combinations of mutations. It contains 23% of the PR1 mutant structures with the mutation L76M (Fig. 9B). These structures represent 88% (36/41) of the structures of this sub-cluster. We noted that two single mutant structures with the mutation L76M, noted PR1_L76M_, belong to this sub-cluster. These results suggest that the mutation L76M is important to the structural change of residue 45_B observed between PR1 and PR2 structures.

This hypothesis is confirmed by the structural comparison of PR1 and PR2 pocket (Fig. 8A). A visualization of PR1 and PR2 pockets showed that residue 45_B is near to substituted residue 76_B in the three-dimensional (3D) space. Thus, we speculated that the amino acid change occurred at position 76_B between PR1 and PR2 might be the origin of the conformational change at residue 45_B between the two PRs. The amino acid change at position 76 corresponds to a substitution of a leucine (in PR1) by a methionine (in PR2), which has a longer side chain. By superimposing PR1 and PR2 pockets (Fig. 8A), we noted that this amino acid change could induce a steric clash between the M76_B (methionine of residue 76 in the chain B) and K45_B in PR1, which is illustrated by the overlap of their van der Waals spheres. To avoid this steric clash, a movement of the side chain of K45_B could occur, resulting in its specific conformation in PR2.

To study in more detail the role of mutation L76M in the structural deformation of residues 45_B, we analysed the five single mutant structures containing the mutation L76M, referred to as PR1_L76M_ and numbered from one to five. We visually compared the conformation of residue 45_B in the five PR1_L76M_ mutants with the residue in wild-type PR1 and PR2 structures (Fig. 8B). Two PR1_L76M_ mutants had a 45_B residue with a conformation close to that observed in the wild-type PR2 structure. This confirms the role of the substitution occurring at position 76 in the structural change observed at position 45_B between the wild-type PR1 and PR2 structures. However, we observed that the M76_B side chain in the five PR1_L76M_ mutants and PR2 had different conformations. This suggests that the substitution L76M could be responsible for the structural change occurring at position 45_B but is not sufficient to explain all structural differences between PR1 and PR2 pocket. According to the three-dimensional structure of PR1, the substituted residue 76_B is spatially close to the substituted residue 32_B. In addition, the classification of mutant and wild-type PR1 and PR2 structures showed that 14% of PR1 mutant structures with the mutation I32V are clustered with wild-type PR2 structure in sub-cluster 3 (Fig. 9B). This indicates that these 23 mutant structures have a residue 45_B conformation closed to this in the wild-type PR2. Most of these structures with the mutation I32V (78%) also contain the mutation L76M. Thus, we speculated that this second substitution (I32V) plays a role in the residue 45_B deformation by modifying the 76_B side-chain conformation.

To test this second hypothesis, we analysed the five PR1 mutants containing the mutations V32I and L76M, referred to as PR1_V32I+L76M_ and numbered from one to five. Figure 8B shows the conformation of residues 45_B, 76_B and 32_B in the five PR1_V32I+L76M_ mutants. We observed that residues 45_B and 76_B had similar conformations in PR1_V32I+L76M_ mutants and in wild-type PR2. In Fig. 8B, we observed an overlap between the van der Waals spheres of the PR1_V32I+L76M_ V32_B residue and the PR1_L76M_ M76_B residue. In addition, we noted an overlap between the van der Waals spheres of the PR1_V32I+L76M_ M76_B residue and the PR1 K45_B residue. Thus, we speculated that substitution V32I in chain B induces a structural change in the M76_B side chain to avoid a steric clash between residues I32_B and M76_B in PR1 mutants. This structural change at the M76_B residue could induce a steric clash between this residue and residue 45_B, which could induce structural changes at residue 45_B.

## Discussion

In this work, we studied the effects of sequence variations between PR1 and PR2 binding pocket. Ligand-binding pockets were extracted from 15 PR1 and 13 PR2 structures (including 4 unbound and 24 bound PR structures complexed with different ligands) by proximity to all bound ligands of the PR set. The advantage of this approach is that the pocket is estimated by taking into account the flexibility and diversity of bound ligands. Thus, the obtained pockets are not dependent on the co-crystallized ligands and therefore allow comparisons of PR1 and PR2 pockets bound to different ligands and not only FDA-approved drugs, as in previous studies^27,28^. We then compared the composition and properties of estimated PR1 and PR2 pockets using a large set of physicochemical and geometric descriptors.

These comparisons showed that both PR1 and PR2 pockets are composed of two types of atoms: (i) atoms conserved across the different PR structures and (ii) flexible atoms. Atoms exhibiting conserved conformations were located in the catalytic site and wall regions, indicating that these regions are important for PR function. This is consistent with results obtained using molecular dynamics analysis, a time-consuming method that highlights the rigidity of the catalytic site^27–30^. In addition, considering pockets extracted from both bound and unbound PR structures, pocket comparison allowed the identification of pocket atoms involved in PR deformation upon ligand binding. As previously reported, PR pocket atoms located in the flap regions undergo structural deformation upon ligand binding. Our results showed that this deformation was also observed at two pocket residues (23 and 32) adjacent to the catalytic site and two residues (81 and 82) adjacent and within the wall region. Our results showed that the flap deformation induced by ligand binding has an impact on the PR pocket properties. Indeed, as several flap residues constituting the pocket tip were missing in unbound pockets, the unbound pockets tended to be smaller and more convex than the bound pockets, with more polar and charged residues. Thus, the comparison of multiple conformations of PR1 and PR2 pockets allowed us to determine relevant information concerning the PR pocket flexibility using a very simple and quick method.

Additionally, the comparison of bound PR1 and PR2 pockets allowed us to characterize the differences in their properties. Bound PR1 and PR2 pockets were more easily differentiated by physicochemical descriptors than geometric descriptors. PR2 pockets were more hydrophobic, with fewer hydrophobic residues, and had a higher number of tiny residues with more oxygen atoms and fewer nitrogen atoms than PR1 pockets. Chen *et al*. (2014) reported that PR1 structures obtained using molecular dynamics simulations had a larger active site volume than molecular dynamics models of PR2^27^. Our results are not in agreement with these findings. This can be explained by the fact that our analysis includes eight different ligands, whereas Chen *et al*.’s study focused only on two FDA-approved drugs, APV and DRV. We speculate that the property changes in PR2 pockets relative to PR1 pockets could modify ligand binding in PR2, which could partially explain the decreased susceptibility of PR2 to most PIs. The classification of PR1 and PR2 bound pockets showed that the pockets extracted from PR2 structures complexed with DRV and APV were similar in terms of the six used properties, while these two similar drugs have different efficiencies against PR2 (EC_50_ = 58 ± 32 nM for DRV and EC_50_ >1000 nM for PR2)^3^. This suggests that property changes that explain the difference between PR1 and PR2 susceptibility to PIs do not explain the difference in PR2 susceptibility to DRV and APV.

We then explored the link between sequence variations between PR1 and PR2 pockets and their physicochemical differences. We demonstrated that the property changes between PR1 and PR2 structures are directly explained by the six substitutions (T31S, V32I, M46I, I47V, L76M, and V82I) located in PR pocket. Indeed, these substitutions modified directly or indirectly the atom composition of PR2 pockets relative to PR1 pockets. For example, these substitutions induce that several atoms were observed in only one PR type; atoms 46_B_CG2 and 82_A/B_CD1 were observed only in PR2 pockets, whereas atoms 47_A/B_CD1 and 76_A/B_CD1 were observed only in PR1 pockets. In addition, our results suggest that side-chain changes occurring at residues 31, 46, and 82 induce structural changes in their main-chain atoms. The differences in atom composition and structural changes between PR1 and PR2 pockets could play a role in the PR2 resistance to PIs by modifying PI interactions with the PR and thus affecting ligand recognition and binding. Priestle *et al*. (1995) has reported the putative role of these substitutions in the alteration of PI recognition by comparing the crystallographic structures of PR1 and PR2 complexed with CGP 53820 inhibitor. In their study, the authors suggested that the larger side chain of residue 82 in PR2 (isoleucine) relative to residue 82 in PR1 (valine) avoid the deformation of the loop 80–83 implied in PI recognition^31^. In addition, the putative role of the substitutions V32I, I47V, and V82I in the alteration of PI interactions with residues 32, 47, and 82 have been previously reported in several studies^3,15,16^. For example, the comparison of the crystallographic structures of PR1 and PR2 complexed with DRV showed that hydrophobic interactions between DRV and PR2 are more elongated than those in PR1-DRV complex and supposed to result from substitutions V32I, I47V, and V82I^15^. Tie *et al*. (2012) compared structural and biochemical features of PR1 (PDB code: 2IEN)^16^, PR2 (PDB code: 3EBZ)^15^, and PR1 mutant with three substitutions (V32I, I47V, and V82I PDB code: 3S43)^16^ complexed with DRV. PR1 mutant and PR2 had similar biochemical properties and DRV had similar interaction network in these two PRs. For example, residue 47_A has two hydrophobic interactions in PR1-DRV complex, whereas it had no contact in PR1 mutant and PR2 complexes that is consistent with our observation that atom 47_A_CD1 is missing in PR2 pocket^16^. This difference at position 47 was not observed during the comparison of other PR1-DRV complex (PDB code: 4DQB)^32^ with the PR2-DRV complex (PDB code: 3EBZ)^15^, but the lack of an interaction between residue 47 with APV was found in the PR2-APV complex (PDB code: 3S45)^16^ but present in the PR1-APV complex (PDB code: 3EKV)^3,33^. This interaction between APV and residue 47 was re-established in the modelled PR2 mutant containing four substitutions I32V, V47I, M76L, and I82V^3^. Differences in interaction network was previously observed for SQV in PR1 and PR1 mutant with three substitutions I32V, V47I, and I82V: in PR1 mutant complex, SQV established more contact with PR1 mutant than with PR1. This is consistent with our results that showed that substitution V82I induced that atom 82_A/B_CD1 is absent in PR1 pockets^16^.

Surprisingly, we observed a large structural change at the non-substituted residue 45 in PR1 and PR2 pockets. This structural change explains some difference between PR1 and PR2 pockets and could be involved in the PR2 resistance to PIs. We observed that residue 45 is spatially close to residues 76 and 32. Using structural comparisons of single and double PR1 mutants—i.e., L76M or V32I + L76M mutants—we suggested that cooperation between substitutions V32I and L76M induces the structural changes at residue 45 in PR2. Indeed, our results showed that substitution at position 32 induces a structural change at position 76 (mutated residue) that also induces structural changes in residue 45. This is in agreement with results obtained by Raugi *et al*. (2016) who showed that the four substitutions I32V, V47I, M76L, and I82V in PR2 cooperate to confer a level of PI susceptibility greater than the sum of each change individually^3^. It would be interesting to validate the putative role of the substitution L76M in the structural deformation of residue 45_B between PR1 and PR2 structures. To do so, the comparison of 45_B residue conformations between crystallographic structures of PR1_L76M_ mutant and wild-type PR1 and PR2 would provide information on the involvement of the substitution L76M in the 45_B residue deformation. However, no PR1_L76M_ mutant structure is available in the PDB. Thus, to validate the putative role of the substitution L76M, the 3D structure of this mutant should be solved. As a perspective, this step could be performed using directed mutagenesis and crystallographic analysis of the recombinant protein obtained with the L76M mutation.

Residue 45_B is not involved in interactions with the ligand, but it is located in the flap region (residues 43–53)^34^, like two substituted residues 46 and 47. It is known that this region undergoes significant deformation upon ligand binding. In addition, molecular dynamics simulations of PR1 and PR2 complexes showed that the regions around residues 40 and 50 exhibit the highest dynamic fluctuation in PR2 relative to PR1^27,28^. Therefore, our results suggest that the T31S, M46I, and V82I amino acid changes induce structural changes in PR2 that can affect PI binding. In addition, mutations V32I and L76M induce structural change at residue 45. This latter structural change coupled with the two substitutions at positions 46 and 47 could modify the PR2 flap flexibility relative to PR1, which could also affect PI binding.

We showed that PR2 pockets are more hydrophobic than PR1 pockets that it is induced by the substitutions V32I, M46I, and V82I. Thus a possible strategy for improving PI efficiency for HIV-2 would be to increase their hydrophobicity to enhance their hydrophobic interactions with PR2 residue 32, 46 and 82. In addition our results showed that three main-chain atoms of substituted residues (31_A_CA, 46_A_O, and 82_B_O) were more frequent in PR2 than in PR1 pockets. Thus, it would be interesting to develop molecules capable of interacting with the PR2 *via* interactions with the backbone oxygen of residues 46 and 82. Favour the backbone interactions between PI and PR2 could also be a strategy to combat against HIV drug resistance, as previously developed for the design of DRV^35^.

## Conclusion

In this study, we explored the effects of the sequence variation between PR1 and PR2 pockets on their properties and structures. We used a large set of descriptors to precisely characterize pockets extracted from 15 PR1 and 13 PR2 structures. This PR set contained unbound and bound forms complexed with different ligands that allowed us to consider the PR pocket flexibility involved in adaptation and recognition of diverse ligands. The comparison of PR1 and PR2 pocket properties using statistical approaches showed that the PR2 pocket is more hydrophobic and contains a higher number of tiny residues than the PR1 pocket. Structural analyses of PR1 and PR2 pockets suggest their amino acid changes (T31S, V32I, M46I, I47V, L76M and V82I) induce structural changes in their substituted and non-substituted residues that modify the physicochemical properties of PR2 pockets. These induced structural changes could affect PI binding and could thus at least partially explain the resistance of PR2 against PIs. In addition, our results suggested an atomic mechanism that corresponded to cooperation between substitutions I32V and L76M, yielding the large structural changes at residue 45 in PR2 relative to PR1. This induced structural change and those induced by substitutions at positions 46 and 47 are located in the flap regions. Thus, our results suggest that these substitutions inside the PR pocket could also impact and modify the PR2 flexibility. This could have effects on the transition between open and semi-closed conformations of PR2 and on PI binding. Our analyses suggest that to improve the PR2 inhibitor efficiency a possible strategy that could consist in increasing the inhibitor hydrophobicity and enhancing their interactions with the backbone atoms of residues 46 and 82.

## Material and Methods

### PR structure

All available PR structures of both HIV-1 and HIV-2 were downloaded from the PDB^20^. From these PDB structures, we selected X-ray PR structures with a resolution smaller than 3 Å and structures with wild-type sequences (structures with experimental mutations to decrease spontaneous proteolysis were considered as wild-type sequences). In addition, for PR1, we retained only structures extracted from HIV-1 subtype B. From this PDB structure set, we retained unbound PR structures and PR1 and PR2 structures complexed with the same ligands. This resulted in a set of 26 PDB files: 15 for PR1 and 11 for PR2. Two PDB files (PDB codes: 2MIP and 1HSH) contained two dimers. For these, we separated the two dimers, which were stored in two PDB files named in our work as 2MIP_AB and 2MIP_CD for the 2MIP PDB file and 1HSH_AB and 1HSH_CD for the 1HSH PDB file. Ultimately, the PR dimer set included 28 PR dimers: 3 unbound PR1 monomers, 1 unbound PR2, 12 bound PR1 and 12 bound PR2 complexed with 8 different ligands (Table S1). For each ligand, we have at least one structure of both PR1 and PR2.

The PR chain names of all PR structures were homogenized, and the first chain of each PDB file was labelled A and the second labelled B. In addition, PR residue numbers in each PDB file were also homogenized; each chain began with residue 1 and ended with residue 99. All structures of unbound PR1 contained only one monomer. To compensate for the lack of PR1 unbound dimer structures in the dataset, artificial dimers of PR1 were generated using a symmetric reflection of PR1 unbound monomer structures to reconstruct dimers using symmetry mates function within 5 Å with the PyMOL software^36^. All structures were then placed in the same 3D referential by superimposing the 28 structures onto the unbound PR2 (PDB code: 1HSI, arbitrarily chosen) using the pymol software^36^. This resulted in a set of 28 PRs, named the PR set.

### Pocket extraction considering the ligand diversity and flexibility

Estimation of the pockets was based on the identification of PR atoms located in close proximity to all ligands of the dataset. To do so, we first superimposed the 24 bound PR structures onto the unbound PR2 (PDB code: 1HSI). As PR is not fully symmetrical despite its homodimeric composition and to compensate for the arbitrary designation of the two chains, two superimpositions were performed for each structure as follows: (i) chain A of the structure was superimposed onto the 1HSI chain A, and (ii) chain B of the structure was superimposed onto the 1HSI chain A. This step yielded a set of 48 superimposed PRs, referred to as the superimposed-PR_AB_ set. Second, all bound ligands of the superimposed-PR_AB_ set were extracted and constituted the superimposed ligand set. This superimposed ligand set accounted for all alternate conformations of all ligands. Then, all these superimposed ligands were placed onto the 28 superimposed structures of the PR set. Finally, pockets were extracted from the 28 superimposed structures by extracting target atoms located at least 4.5 Å from all superimposed ligands. These pockets corresponded to the largest pocket capable of binding available PR ligands.

This pocket-estimation protocol allowed the extraction of the ligand-binding pocket from a target structure independent of the protein deformation due to each specific ligand. This approach has several advantages: (i) it estimates a consensus binding pocket by considering the diversity and flexibility of various bound ligands; (ii) as all available ligands are considered during the estimation, the shape of the estimated pockets is not affected by the co-crystallized ligand’s shape; and (iii) it can be used to estimate pockets from unbound structures of a target. Using this approach, we estimated 28 pockets extracted from unbound and bound PR structures.

### Descriptors used to characterize pocket properties

PR pockets were described using a set of 51 physicochemical and geometric descriptors^22^; see S1 Appendix. Fourteen descriptors characterized the geometry of the pockets, specifically the volume of the pockets, their shape, their convexity, and their sphericity, and three corresponded to their inertia moments. The 37 remaining descriptors characterized the physicochemical properties of the pockets. They described the atom composition of the pockets, their composition in residues, and their global properties, such as their hydrophobicity, polarity, and charge. Several descriptors were dedicated to quantifying the pocket hydrophobicity; *p_hydrophobic_residues* quantified the proportion of hydrophobic residues in the pocket, *hydrophobicity_kyte* quantified the global pocket hydrophobicity based on residue properties^37^, and *hydrophobicity_pocket_pocket* quantified the global pocket hydrophobicity based on the solvent accessibility computed using NACCESS^38^. The three descriptors provided complementary information. For example, a pocket can have a high *hydrophobic_kyte* value but a small *p_hydrophobic_residues* value, indicating that this pocket is globally very hydrophobic despite having only a few hydrophobic residues.

From these descriptors, we then removed nine descriptors with null variance in the PR pocket set. Ultimately, the PR pockets were described using 42 physicochemical and geometric descriptors.

### Visualization of the pocket space

To visualize the space sampled by the pockets, we performed principal component analysis (PCA) based on the 42 physicochemical and geometric pocket descriptors. The PCA was computed using the FactoMineR package in R^39^. PCA projects standardized data—i.e., the pocket descriptors—into a subspace made of orthogonal linear combinations of pocket descriptors, so-called principal components. Data may then be explored in a smaller dimensional space spanning the most informative view, according to the data variability.

### Statistical selection of the most informative descriptors for distinguishing PR1 and PR2 pockets

#### Random Forest Training

Random forest (RF) model is a supervised, non-parametric method of classification^40^. RF consists of a collection of decision trees, wherein each tree is obtained from a subset of the data. We trained and analysed a RF classification model to identify descriptors allowing the discrimination between PR1 and PR2 bound pockets. It was trained on bound PR pockets using their 42 physicochemical and geometric descriptor values. The RF model was computed using the RandomForest package within R^41,42^. The S2 Appendix shows the different steps for the training and analysis of the RF model.

#### Selection of descriptors differentiating PR1 and PR2 pockets

We selected descriptors allowing the differentiation of PR1 and PR2 pockets using the computation of their importance in the *RF*_*PR1-PR2*_ model (S2 Appendix). The importance quantifies the involvement of a descriptor in the *RF*_*PR1-PR2*_ model. The higher the importance value is, the better the descriptor in separating PR1 and PR2 pockets. In a second step, we tested the descriptor robustness for separating PR1 and PR2 pockets by computing the *fMVI* parameter, which quantifies the number of models that select this descriptor as important among the 500 computed RF models (S2 Appendix). The higher the *fMVI* of a descriptor is, the more robust the descriptor is to differentiate PR1 and PR2 pockets. In a third step, we tested the statistical significance of the selected important descriptors by computing the importance p-value, denoted the *pvalue*_*IMP*_, of each descriptor—i.e., the probability that descriptor importance was higher in a random RF model than in the *RF*_*PR1-PR2*_ model^43^. A descriptor with a *pvalue*_*IMP*_ less than 0.05 was considered significant for separating PR1 and PR2 pockets. A descriptor was determined to be important for separating PR1 and PR2 pockets if it had an importance score in the *RF*_*PR1-PR2*_ model higher than 0.5, a fMVI value higher than 90% and a *pvalue*_*IMP*_ smaller than 0.05.

### PR1 and PR2 pocket classification

PR pocket classification was established using the same protocol as in^23^, based on the *p* most important pocket descriptors for separating PR1 and PR2 pockets selected using the *RF*_*PR1-PR2*_ model. Euclidean distances, denoted as *ED*_*pock*_, between all PR1 and PR2 pockets were computed based on the scaled distances obtained with the *p*-selected descriptors. These distances quantified the similarity between each PR pocket pair. A short distance separated two similar pockets, whereas two dissimilar pockets were separated by a long distance. From these *ED*_*pock*_ distances between all PR pockets, a hierarchical classification of the PR pocket set was computed using Ward method aggregation, which creates a dendrogram of PR1 and PR2 pockets according to the set of important descriptors previously identified.

### Modelling of mutant PR1 structures

The PR1 mutant structures were modelled using the FoldX suite^26^ that includes different subroutines allowing for example the rearrangement of side chains to reduce the energy content of a protein structure (RepairPDB command), introducing mutations and optimizes the structure of the mutant (BuildModel command), and calculating the ΔG to fold the proteins from their unfolded state (Stability command). FoldX is a frequently used algorithm to predict the protein stability upon mutations and to model a protein mutant structure. For example, Marjan *et al*. (2014) modelled the structure of 37 COX-1 structures using FoldX to analyse the functional impacts of 37 nsSNPs on aspirin inhibition potency of COX-1^44^. Buß *et al*. (2018) used Fold-X to predict the ΔΔG value of 8246 single-site mutations of ω-Transaminase to identify the most thermostable variant^45^. See Buß *et al*. (2018) for a review of the FoldX application^46^.

The core function of FoldX, the empirical force field algorithm, is based on free energy (ΔG) terms aiming to calculate the change of ΔG in kcal mol−1. It is composed of a solvation term, a van der Waals term, H-bond, and electrostatic terms and entropic terms for the backbone and side chains^26^. FoldX force field has been validated on a blind-test database of 625 single-point mutants in 27 simple proteins resulting in a correlation coefficient of 0.73 between predicted and experimental ΔΔG of folding. Several studies had tested the performances of softwares dedicated to the prediction of protein stability^45,47,48^. For example, Khan and Vihinen (2010) showed that FoldX were one of the most reliable predictors on a set of 1784 single mutations found in 80 proteins with 64% of well prediction^47^. See Buß *et al*. (2018) for a review of the FoldX accuracy^45^.

To construct PR1 mutant structures, we used the BuildModel command of FoldX suite. This command introduces a mutation on the wild-type structure using a side-chain rotamer library. It then optimizes the configurations of the side chains of amino acids in the vicinity of the mutated residue. In FoldX, the mutated residue vicinity was defined as residues for which their half-spheres were situated at less 5 Å from the mutated residue half-sphere. The half-sphere of a residue is proportional to the mobility and length of its side chain.

In this study, we constructed 3D structures of PR1 mutants based on the wild-type PR1 structure complexed with APV (PDB code: 1HPV) as a template. First, the APV ligand, metal atoms, and water molecules were removed from the 1HPV structures. We built the 3D structure of PR1 mutants using the BuildModel command of FoldX^26^. Each model was generated with five runs, and other options were set to default. PR1 mutant structures were superimposed onto the unbound PR2 structure (PDB code: 1HSI).

### Comparison of the conformation of residue 45_B in wild-type and mutant PR structures

To compare the conformation of residue 45_B in all PR1 mutant structures and the wild-type PR1 and PR2 structures, we first computed the Root Mean Square deviation (RMSD) between residue 45_B (i) of all PR1 mutant structure pairs, (ii) between the PR1 mutant structures and the wild-type PR1 (PDB code: 1HPV) and, (iii) between the PR1 mutant structures and the wild-type PR2 (PDB code: 3S45) structures. These RMSD were computed using the 9 atoms of the lysine in the 45_B position (N, CA, C, O, CB, CG, CD, CE, NZ). We obtained an RMSD-matrix with 317 columns and 317 rows (=315 PR1 mutant +1 wild-type PR1 +1 wild-type PR2 structures). Using this RMSD matrix and Ward method aggregation, we then computed a hierarchical classification of the 315 PR1 mutants, 1 wild-type PR1 and 1 wild-type PR2 structures. From this classification, we extracted four clusters by cutting the obtained dendrogram with a height of 2. We then determined, for each cluster, the percentage of mutations that it contains, i.e., for a given mutation (e.g. L76M) and a given cluster (e.g. cluster 3), the number of mutant structures in cluster 3 with the mutation L76M divided by the number of mutant structures in cluster 3.

## Electronic supplementary material

Supplementary Information
