# Supplementary material for: Exploration of the effect of sequence variations located inside the binding pocket of HIV-1 and HIV-2 proteases
Source: Sci Rep. 2018 Apr 10;8:5789. doi: 10.1038/s41598-018-24124-5 (PMC5893546; doi:10.1038/s41598-018-24124-5)
Supplement: Supplementary file 1 — Supplementary Information [file 41598_2018_24124_MOESM1_ESM.pdf]

Supporting Information

Title: Exploration of the effect of sequence variations located inside the binding pocket of HIV-1 and HIV-2 proteases

Authors: Dhoha Triki[1,3], Telli Billot[1,3], Benoit Visseaux[2,3], Diane Descamps[2,3], Delphine Flatters[1,3], Anne-Claude Camproux[1,3] & Leslie Regad[1,3]*

Affiliations:

[1] Molécules thérapeutiques *in silico* (MT*i*), INSERM UMR-S973, Paris, France

[2] IAME, UMR 1137, INSERM, Laboratoire de Virologie, Hôpital Bichât, AP-HP, Paris, France

[3] Université Paris Diderot, Sorbonne Paris Cité, Paris, France

* corresponding author: leslie.regad@univ-paris-diderot.fr

S1 Table. Description of the PR set.

	PR1 (15)		PR2 (13)	
Ligand	PDB code	Resolution (Å)	PDB code	Resolution (Å)
none	1HHP	2.70	1HSI	2.50
	2HB4	2.15		
	3PHV	2.70		
APV	1HPV	1.90	3S45	1.51
	3EKV	1.75		
	3NU3	1.02		
IDV	1SDT	1.30	1HSH_AB	1.90
			1HSH_CD	
DRV	2IEN	1.30	3EBZ	1.2
	4HLA	1.95		
	4LL3	1.95		
GRL (DRV derivative)	2HB3	1.35	3EC0	1.18
065 (DRV derivative)	2Z4O	1.60	3ECG	1.18
C20	1HIH	2.20	1HII	2.3
1ZK	1HIV	2.00	1IVP	2.50
Peptides	2NPH	1.65	2HPE	2.00
			2HPF	3.00
			2MIP_AB	2.20
			2MIP_CD	

S1 Appendix. List of descriptors used to characterize PR1 and PR2 pockets.

In this study, PR1 and PR2 pockets were characterized using a set of 51 physicochemical and geometric descriptors [1].

- Fourteen descriptors characterized the geometry of the pockets:
    - 3 quantified pocket volume: *VOLUME_HULL*, *FACE*, and *SMALLEST_SIZE*.
    - 4 quantified the pocket shape: *RADIUS_HULL*, *SURFACE_HULL*, *DIAMETER_HULL*, and *RADIUS_CYLINDER*.
    - 3 quantified the pocket convexity: *PCI*, *%_ATOM_CONVEX,* and *CONVEX_SHAPE_COEFFICIENT*.
    - 1 quantified the pocket sphericity: *PSI*.
    - 3 corresponded to the inertia moments: *INERTIA_1*, *INERTIA_2*, and *INERTIA_3*.
- Thirty-seven descriptors characterized the physicochemical properties of pockets:
    - 24 described the atom composition: *C_ATOM*, *p_side_chain_atom*, *p_main_chain_atom*, *p_N_atom*, *p_ND1_atom*, *p_NE2_atom*, *p_nitrogen_atom*, *p_Nlys_atom*, *p_Ntrp_atom*, *P_C_atom*, *p_carbone_atom*, *p_Car_atom*, *p_Ccoo_atom*, *p_Carg_atom*, *p_Cgln_atom*, *p_O_atom*, *p_oxygen_atom*, *p_Ooh_atom*, *p_Otyr_atom*, *p_Ocoo_atom*, *p_S_atom*, *p_sulfur_atom*, *p_hyd_atom*, and *p_hydrophobic_atom*.
    - 9 described the pocket residue composition: *p_tiny_residues*, *p_small_residues*, *p_hydrophobic_residues*, *p_polar_residue*, *p_charged_residues*, *p_negative_residues*, *p_positive_residues*, *p_aliphatic_residues*, and *p_aromatic_residues*.
    - 4 described the global properties of the pocket: *polarity_pocket_pocket*, *hydrophobic_kyte*, *hydrophobicity_pocket_pocket*, and *charge*.

S2 Appendix. Study of the robustness of the performance of the random forest model

Methods

Random forest model definition. Random forest (RF) model [2] is a supervised, non-parametric method of classification. RF consists of a collection of decision trees obtained from a subset of the data. First, when constructing a tree classifier, a bootstrap sample is drawn from the original observations. The samples that are not in the bootstrap sample are called out-of-bag (OOB) data. Second, a subset of explanatory variables is randomly selected. Each node of the individual classification trees is trained on a bootstrap sample of observations described by a randomly selected variable subset. The class of a new sample is determined by the majority of the votes of all trees in the forest.

Training of the RF model. RF model was computed using the bound PR pockets in order to predict the type of pockets, either PR1 or PR2 pockets. This was accomplished using their 42 physicochemical and geometric descriptor values and the randomForest package within R [3,4]. The *ntree*—i.e., number of trees in the forest—and *mtry*—i.e., number of variables randomly sampled as candidates at each split —*values* were fixed at 200 and 12, respectively, after an optimization step using a grid approach. Thus, the generation of each tree tested 12 pocket descriptors at each split and was generated to create nodes with the best classification of the training sample. The process was repeated to generate 200 trees, which made up the forest, named $RF_{PR1\text{-}PR2}$.

***$RF_{PR1\text{-}PR2}$* model performance**. The performance of the RF model was quantified using the OOB

error rate, which reports the proportion of samples that are classified incorrectly on average across the trees using the OOB sample. As the RF model is a machine-learning approach based on a double-random process, we tested the robustness of the $RF_{PR1\text{-}PR2}$ model's performances. We computed the OOB errors of 500 RF models, named $RF^{500}_{PR1-PR2}$ models, trained using the same data used to train the $RF_{PR1\text{-}PR2}$ model. In a second step, we compared the $RF_{PR1\text{-}PR2}$ model's performance to the performance of random RF models. We computed 500 RF models, named $RF_{permuted}$ models, trained using permuted data. First, the values of the response variable, here PR1 or PR2, were randomly permuted. Using this permuted pocket and the 42 descriptor values, a new RF model, named $RF_{permuted}$, was computed. The overall performance of the $RF_{permuted}$ model was then computed. This procedure was repeated 500 times.

We considered the $RF_{PR1\text{-}PR2}$ model to be performing and robust if this model and the set of 500 models had a weak OOB error value. In addition, their performances must have been significantly better than the $RF_{permuted}$ model performances.

Selection of descriptors differentiating PR1 and PR2 pockets. In a second step, we analysed the $RF_{PR1\text{-}PR2}$ model to select descriptors that accurately separated PR1 and PR2 pockets. This selection was based on the importance score of each descriptor. The importance score corresponded to the average decrease in model accuracy on the OOB samples when the values of the respective descriptor were randomly permuted [2]. Large values of the mean decrease in accuracy indicate that the descriptor is important for classifying the sample correctly. From the $RF_{PR1\text{-}PR2}$ model, we selected the descriptors having the highest importance score values.

To test the robustness of the important descriptor selection, we analysed the most important descriptors in the $RF^{500}_{PR1-PR2}$ models. We selected the ten descriptors with the highest importance scores in each $RF^{500}_{PR1-PR2}$ model. Then, we computed the fraction of models wherein the descriptor

was selected as one of the ten most important descriptors, named *fMVI*, for each descriptor. The important descriptor selection is robust if most models yield the same important descriptors.

In the final step, we analysed the significance of the selected important descriptors by computing the importance p-value for each descriptor [5]. We computed the importance score of each descriptor in the 500 $RF_{permuted}$ models. This led to a vector of 500 importance measures for every variable, which we called the null importances. We then computed a non-parametric estimation of the importance p-value, named the $pvalue_{IMP}$, by determining the fraction of null importances that were more extreme than the importance of the $RF_{PR1\text{-}PR2}$ model. A descriptor was selected as important for separating PR1 and PR2 pockets if it had an importance score in the $RF_{PR1\text{-}PR2}$ model higher than 0.5, a fMVI value higher than 90% and a $pvalue_{IMP}$ smaller than 0.05.

Results

Training of RF model. To identify the most important descriptor for separating PR1 and PR2 bound pockets, we trained an RF model on the 24 bound pockets characterized by the 42 descriptors. The obtained RF model, named $RF_{PR1\text{-}PR2}$, exhibited an OOB error rate of 0.04. We tested the robustness and the validity of the $RF_{PR1\text{-}PR2}$ model by comparing the performances of $RF_{PR1\text{-}PR2}$ with those of the $RF^{500}_{PR1-PR2}$ and $RF_{permuted}$ models (Table S2). We observed that $RF^{500}_{PR1-PR2}$ models also exhibited very good performances, with OOB error rates ranging from 0 to 0.08 with an average error of 0.003 ± 0.011. In contrast, the $RF_{permuted}$ models showed bad performances with very large average OOB error values (0.54 ± 0.13). Thus, we concluded that the $RF_{PR1\text{-}PR2}$ model clearly had higher performances than random models. This result highlights the relevance of the $RF_{PR1\text{-}PR2}$ model for discriminating PR1 and PR2 bound pockets according to their descriptors.

Data	Models	OOB error
Initial data of 42 physicochemical and geometric descriptors computed for 24 PR bound pockets.	$RF_{PR1\text{-}PR2}$ model	0.04
	500 RF models	0.003 ± 0.01
Permuted data	$RF_{permuted}$ models	0.54 ± 0.13

Table S2: Performances of the different computed RF models.

Selection of descriptors separating PR1 and PR2 bound pockets

Figure 1 presents the involvement of the 42 descriptors in the $RF_{PR1\text{-}PR2}$ model, quantified by their importance scores.

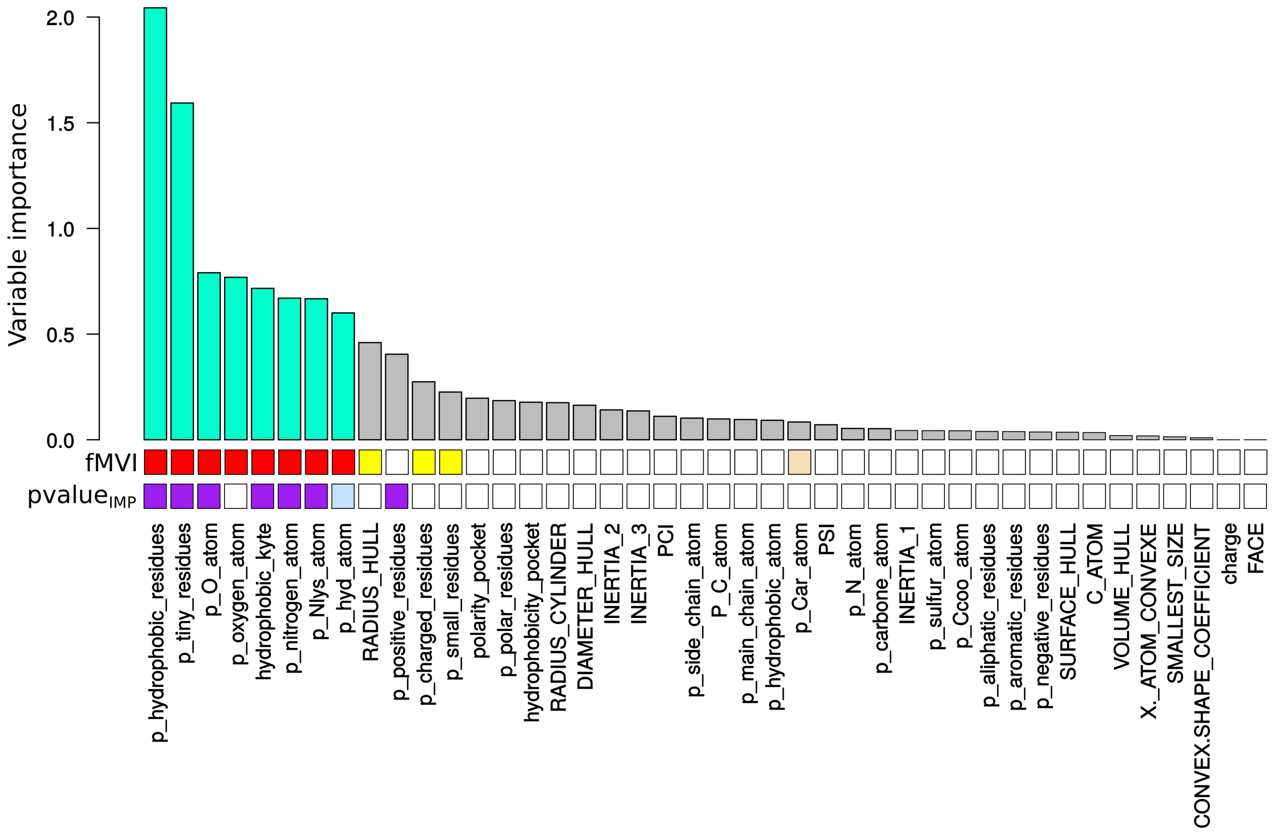

Figure 1 : RF importance scores of the 42 pocket descriptors in the $RF_{PR1\text{-}PR2}$ model. The eight descriptors selected as important for the RF model (with an importance score higher than 0.5) are coloured green. fMVI corresponds to the proportion of RF models where a variable was selected as one of the ten most important variables. This value was computed from a simulation of 500 RF models computed using the same data and the same parameters as those used to compute the $RF_{PR1\text{-}}$

$_{PR2}$ model (see S2 appendix). fMVI values between 0% and 20% are shown in white; fMVI values between 20% and 40% are shown in wheat; fMVI values between 40% and 60% are shown in yellow; fMVI values higher than 60% and 90% are shown in magenta; and fMVI values higher than 90% are shown in red. $pvalue_{IMP}$ corresponds to the importance score p-value, computed as a fraction of null importance (importance computed from $RF_{permuted}$) scores that were more extreme than the importance score in the $RF_{PR1-PR2}$ model. All $pvalue_{IMP}$ values smaller than 0.06 are coloured light blue, and $pvalue_{IMP}$ values smaller than 0.05 are coloured purple.

Among the 42 pocket descriptors involved in the $RF_{PR1-PR2}$ model, eight had an importance score higher than 0.5: *p_hydrophobic_residues*, *p_tiny residues*, *p_O_atom*, *p_oxygen_atom*, *hydrophobibity_kyte*, *p_nitrogen_atom*, *p_Nlys_atom*, and *p_hyd_atom* (Figure RF-desc). We noted that these eight descriptors were selected as the most important descriptors in 95% of the $RF^{500}_{PR1-PR2}$models (*fMVI* > 95%). This result indicates that the selection step of the important descriptors was robust. In addition, six of them had a significant p-value ($pvalue_{IMP}$ < 0.05) (Figure fig:RF-desc). These results collectively show that these six descriptors are able to separate PR1 and PR2 pockets. They characterize the pocket hydrophobicity (*p_hydrophobic_residues* and *hydrophobibity_kyte*), the composition of certain atoms (oxygen [*p_oxygen_atom*] and nitrogen [*p_nitrogen_atom* and *p_Nlys_atom*]) and tiny residues (*p_tiny residues*).

References

1. Borrel A, Regad L, Xhaard H, Petitjean M, Camproux A-C. PockDrug: A Model for Predicting Pocket Druggability That Overcomes Pocket Estimation Uncertainties. J Chem Inf Model. 2015;55: 882–895. doi:10.1021/ci5006004
2. Breiman, L. Machine Learning. 2001;45: 5-32. https://doi.org/10.1023/A:1010933404324
3. Svetnik V, Liaw A, Tong C, Christopher Culberson J, Sheridan RP, Feuston BP. Random Forest: A Classification and Regression Tool for Compound Classification and QSAR Modeling. J Chem Inf Comput Sci. 2003;43: 1947–1958. doi:10.1021/ci034160g
4. Liaw a, Wiener M. Classification and Regression by randomForest. R news. 2002;2: 18–22. doi:10.1177/154405910408300516
5. Altmann A, Toloşi L, Sander O, Lengauer T. Permutation importance: A corrected feature importance measure. Bioinformatics. 2010;26: 1340–1347. doi:10.1093/bioinformatics/btq134
